# Whole blood transcriptional profiling in ankylosing spondylitis identifies novel candidate genes that might contribute to the inflammatory and tissue-destructive disease aspects

**DOI:** 10.1186/ar3309

**Published:** 2011-04-07

**Authors:** Fernando M Pimentel-Santos, Dário Ligeiro, Mafalda Matos, Ana F Mourão, José Costa, Helena Santos, Anabela Barcelos, Fátima Godinho, Patricia Pinto, Margarida Cruz, João E Fonseca, Henrique Guedes-Pinto, Jaime C Branco, Matthew A Brown, Gethin P Thomas

**Affiliations:** 1 CEDOC, Faculdade de Ciências Médicas da Universidade Nova de Lisboa, Campo dos Mártires da Pátria, n° 130, 1169-056 Lisboa, Portugal; 2 Instituto de Biotecnologia e Bioengenharia, Centro de Genómica e Biotecnologia, da Universidade de Trás-os-Montes e Alto Douro (IBB/CGB - UTAD), Quinta dos Prados, 5000-262 Vila Real, Portugal; 3 Centro Hospitalar Lisboa Ocidental (CHLO), Hospital de Egas Moniz EPE, Rua da Junqueira, n° 126, 1349-019 Lisboa, Portugal; 4 Centro de Histocompatibilidade do Sul, Alameda das Linhas de Torres, n° 117, 1769 - 001 Lisboa, Portugal; 5 Universidade de Trás-os-Montes e Alto Douro, Quinta dos Prados, 5000-262 Vila Real, Portugal; 6 Centro Hospitalar do Alto Minho (CHAM), Hospital Conde de Bertiandos EPE, Largo Conde de Bertiandos, 4990-041 Ponte de Lima, Portugal; 7 Instituto Português de Reumatologia (IPR), Rua da Beneficência, n° 7, 1050-034 Lisboa, Portugal; 8 Centro Hospitalar Baixo Vouga, Hospital Infante D. Pedro EPE, Avenida Artur Ravara, 3814-501 Aveiro, Portugal; 9 Hospital Garcia de Orta EPE, Av. Torrado da Silva, Pragal, 2801-951 Almada, Portugal; 10 Centro Hospitalar de Vila Nova de Gaia/Espinho EPE, Rua Dr. Francisco Sá Carneiro, 4400-129 Vila Nova de Gaia, Portugal; 11 Centro Hospitalar Oeste Norte, Centro Hospitalar das Caldas da Rainha, Rua Diário de Notícias, 2500-176 Caldas da Rainha, Portugal; 12 Unidade de Investigação em Reumatologia, Instituto de Medicina Molecular (IMM), Faculdade de Medicina da Universidade de Lisboa, Edifício Egas Moniz, Av. Professor Egas Moniz, 1649-035 Lisboa, Portugal; 13 Centro Hospitalar de Lisboa Norte, Hospital Santa Maria EPE, Av. Professor Egas Moniz, 1649-035 Lisboa, Portugal; 14 University of Queensland Diamantina Institute, Princess Alexandra Hospital, Woolloongabba, QLD 4102, Australia

## Abstract

**Introduction:**

A number of genetic-association studies have identified genes contributing to ankylosing spondylitis (AS) susceptibility but such approaches provide little information as to the gene activity changes occurring during the disease process. Transcriptional profiling generates a 'snapshot' of the sampled cells' activity and thus can provide insights into the molecular processes driving the disease process. We undertook a whole-genome microarray approach to identify candidate genes associated with AS and validated these gene-expression changes in a larger sample cohort.

**Methods:**

A total of 18 active AS patients, classified according to the New York criteria, and 18 gender- and age-matched controls were profiled using Illumina HT-12 whole-genome expression BeadChips which carry cDNAs for 48,000 genes and transcripts. Class comparison analysis identified a number of differentially expressed candidate genes. These candidate genes were then validated in a larger cohort using qPCR-based TaqMan low density arrays (TLDAs).

**Results:**

A total of 239 probes corresponding to 221 genes were identified as being significantly different between patients and controls with a *P*-value <0.0005 (80% confidence level of false discovery rate). Forty-seven genes were then selected for validation studies, using the TLDAs. Thirteen of these genes were validated in the second patient cohort with 12 downregulated 1.3- to 2-fold and only 1 upregulated (1.6-fold). Among a number of identified genes with well-documented inflammatory roles we also validated genes that might be of great interest to the understanding of AS progression such as *SPOCK2 *(osteonectin) and *EP300*, which modulate cartilage and bone metabolism.

**Conclusions:**

We have validated a gene expression signature for AS from whole blood and identified strong candidate genes that may play roles in both the inflammatory and joint destruction aspects of the disease.

## Introduction

Ankylosing spondylitis (AS) is a chronic inflammatory rheumatic disease characterised by inflammation that leads to bone resorption and bone formation, ultimately resulting in progressive ankylosis [1]. Although the aetiopathogenesis of AS is not yet clearly defined, both susceptibility to and severity of this disease are highly heritable. The major gene association is with the MHC I gene *HLA-B27 *with 95% of patients positive for this gene [2-4]. However, only approximately 5% of *HLA-B27 *carriers suffer from AS, meaning other genes are involved in disease susceptibility. In fact, twin and family studies have suggested that *HLA-B27 *accounts for less than 40% of the overall risk for AS [2,4]. In recent years genetic-association studies have identified several new genes in association with AS. Some of these genes appear specific for AS, whereas others have pleiotropic associations [5,6]. Nevertheless, the mechanism by which *HLA-B27 *and other more recently identified genetic factors involved in AS susceptibility, lead to disease remains uncertain.

Genetic studies provide little information as to the gene activity changes occurring during the disease process. Gene-expression profiling confers a "snapshot" of cellular activity providing information on mechanisms mediating disease changes, elucidating possible pathways involved and can also generate diagnostic gene sets. In AS and spondyloarthritis (SpA) a number of recent studies have defined transcriptional profiles generated from peripheral blood mononuclear cells (PBMCs) isolation requiring immediate sample processing, which is not suitable for larger multicentre studies and limits the viability of such an approach [7]. An alternate approach is to use whole blood samples collected using PAXgene technology which preserves the integrity of the RNA even with limited storage at room temperature allowing delays in transport and handling to occur with minimal RNA degradation [7].

In the current study, we undertook a whole-genome microarray approach to identify a genomic profiling in a Portuguese case-control collection, using RNA from peripheral blood collected using the PAXgene collection system, and validated these gene-expression changes in an independent larger sample cohort using quantitative PCR (qPCR). Our goal was to test whether genomic profiling in such cases, using the more practical PAXgene Blood RNA System^®^, could distinguish AS cases from healthy controls, and identify genomic pathways likely to be involved in AS pathogenesis.

## Materials and methods

### Study subjects

The microarray-based discovery study was performed using samples from 18 AS patients, diagnosed according to the modified New York criteria [8], and 18 gender- and age-matched healthy controls (±5 years). Included patients had Bath Ankylosing Spondylitis Disease Activity Index (BASDAI) scores >4 and Bath Ankylosing Spondylitis Functional Index (BASFI) scores >4. All patients were receiving only NSAIDs and/or sulphasalazine. No TNF, corticoid or methotrexate treated patients were included. Details of the study subjects are shown in Supplementary Table S1 in Additional file 1.

Candidate genes were validated in a second larger cohort of another 78 AS patients and 78 age and sex matched controls (full details in Supplementary Table S2 in Additional file 2).

Peripheral blood samples were collected into PAXgene Blood RNA System^® ^tubes (Qiagen, Doncaster, VIC, Australia) and stored according to the manufacturer's recommendations [9]. This study was approved by the Ethics Committees of the participating centres, and written informed consent was obtained from the individuals involved in this study.

### RNA processing and array analysis

Total RNA was extracted from whole blood samples according to the standard PAXgene protocol, quantified and the integrity assessed by Agilent 2100 BioAnalyser (Agilent, Santa Clara, CA, USA). Only samples with a RNA integrity number above 7.5 were used. To minimize the effects of Globin RNA transcript over-representation, samples were processed with Ambion GLOBINclear^® ^(Applied Biosystems, Mulgrave, VIC, Australia) according to the manufacturer's protocol. cRNA was generated from 500 ng of total RNA using the Illumina TotalPrep cRNA Amplification Kit^® ^(ABI) and hybridized to Human HT-12 V3 Expression BeadChips (Illumina, San Diego, CA, USA). Array data were processed using the Illumina GenomeStudio software, transformed by variance stabilization transformation (VST) [10] and normalized by robust spline normalization [11] using Lumi [12]. Quality control using principal components analysis showed four samples to be outliers and further investigation revealed technical issues with the processing of these samples and thus they were excluded from the analysis.

Gene expression analysis was performed in BRB-ArrayTools [13]. Differentially expressed genes were identified by unpaired t-test with multivariate permutation correction. Gene ontology analysis was carried out in BRB ArrayTools using a LS permutation test which finds gene sets that have more genes differentially expressed among the classes than it would be expected by chance using 100,000 random geneset permutations to compare to the chosen geneset.

### Candidate gene validation using quantitative reverse transcription polymerase chain reaction

Candidate genes were identified from the array studies based upon their fold-change between AS and control, the *P*-value of this difference and their potential biological relevance to AS. Candidate genes were assayed using real-time quantitative PCR-based (qPCR) predesigned TaqMan Low Density Array Cards (TLDA). The TLDA cards had 48 predesigned Taqman qPCR assays, which utilise MGB probes with FAM dye, arrayed in a 384-well format allowing four samples to be assayed per TLDA. Forty-seven candidate genes selected from the whole genome arrays together with a housekeeping gene (*18s*) were arrayed. cDNA was generated from 1 μg of total RNA using the Bioline cDNA synthesis Kit (Bioline, London, UK) according to manufacturer's instructions. qPCR was carried out using SensiMix dT RT-PCR reagent (Quantace, Sydney, QLD, Australia) under the following conditions; 50°C for 2 minutes, 95°C for 10 minutes, and 40 cycles of 95°C for 15 s and 60°C for 60 s [14].

Data were normalized using the housekeeping gene, *18S*, included on the card and quantified using the 2^-ΔCT^[15]. Data were analysed with the Mann-Whitney test and *P-*values <0.05 were considered significant (SPSS v17.0, Chicago, IL, USA).

## Results

### Differential gene expression in AS patients and controls

**Figure 1:**
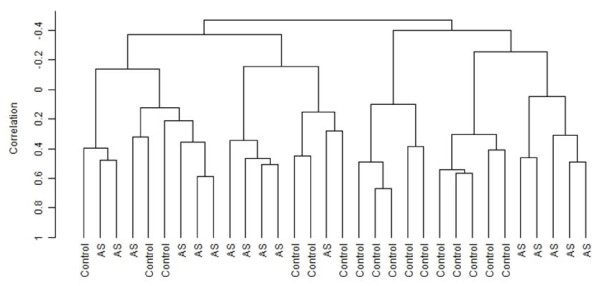
**Clustering on top 3% most variable genes**. Unsupervised hierarchical clustering based upon the top 3% most variable genes (585 genes) showed good delineation between the patient and control samples with six controls and five AS samples misclassifying. The non-perfect classification suggests non-disease related factors also drive the gene expression patterns.

From a total of more than 48,000 probes on the array, 18,159 were found to be expressed in at least one sample and were included for analysis. To estimate the degree of gene expression variation driven by disease status, we undertook unsupervised hierarchical clustering using the top 3% most variably expressed genes without reference to disease status. Clustering with this non-biased geneset gave good delineation between the controls and AS patients with only six controls and five AS patients misclassifying (Figure 1). To identify the genes specifically differentially expressed between patients and controls, we carried out an unpaired T-test corrected for multiple comparisons. A total of 648 probes were considered significantly differentially expressed (80% confidence level of false discovery rate, with 10% false positives) with 204 of these probes (corresponding to 190 genes) having *P*-values <0.0005 (Supplementary Table S3 in Additional file 3). The magnitude of differential expression observed was generally low, with only three genes showing a significant fold-change >2 (with the maximum fold-change being 2.21) and 29 genes >1.5 fold-change. Of the 204 probes, 89 probes were upregulated and 115 downregulated. Our microarray data are available in a public repository, Gene Expression Omnibus (GEO), with the accession number [GEO:GSE25101].

We then selected 47 of the differentially expressed genes for validation by qPCR (Table 1) based upon their *P*-value, fold-change and biological relevance.

**Table 1:** Selected genes for validation by qPCR

Gene symbol	Fold-change (AS/Cont)	Parametric *P*-value
CX3CR1	0.58	3.45E-04
DGKQ	0.62	1.45E-05
SPOCK2	0.65	3.07E-04
SBK1	0.66	1.69E-04
GZMM	0.67	3.39E-04
CDC25B	0.67	2.00E-04
CLSTN1	0.68	4.53E-04
ITGB7	0.68	5.63E-04
PTPN1	0.69	1.50E-06
EP300	0.70	1.26E-04
DOCK10	0.70	1.73E-04
MAPK8IP3	0.70	3.37E-04
BCL11B	0.70	5.88E-04
DNMT1	0.71	6.46E-05
XPC	0.71	5.10E-06
PPP2R1A	0.71	1.73E-04
IL27RA	0.72	4.40E-06
MCM3	0.73	4.24E-04
SYF2	1.34	5.94E-04
PPP2R3C	1.36	1.07E-03
NGFRAP1	1.37	1.87E-04
ZMAT2	1.38	1.58E-04
MYL6	1.41	7.94E-05
S100A8	1.43	6.09E-04
GMFG	1.43	3.91E-05
VAMP5	1.44	4.21E-04
CKLF	1.46	4.30E-04
SHFM1	1.47	8.62E-04
ATG3	1.49	6.10E-04
MRPS18C	1.49	5.71E-04
CLEC4D	1.52	3.47E-04
UBL5	1.52	8.93E-05
AIF1	1.52	5.19E-04
PDCD10	1.54	2.45E-03
NDUFS4	1.54	5.93E-04
SF3B14	1.54	2.39E-04
HMGB2	1.57	3.41E-04
UQCRB	1.63	2.33E-04
TXN	1.68	4.39E-05
CMTM2	1.74	4.78E-04
CIP29	1.75	1.38E-04
CHMP5	1.78	6.80E-04
PSMA4	1.80	8.45E-05
NDUFB3	1.88	1.25E-04
LSM3	1.99	6.72E-04
GNG11	2.15	1.41E-04
CCDC72	2.21	9.64E-05

Genes (*n *= 47) for validation by qPCR were selected from the 648 probes significantly differentially expressed in microarrays based upon their *P*-value, fold-change and biological relevance. AS, Ankylosing spondylitis; qPCR, quantitative polymerase chain reaction.

### Quantitative RTPCR validation

Expression levels of the 47 selected genes were confirmed by qPCR in a second sample set consisting of 78 patients and 78 age and gender matched controls. A total of 28 of the 47 genes showed a similar trend in differential expression between AS and control samples as the array data. Of these, 14 of the 47 considered genes were validated with significant *P*-values with 13 downregulated 1.4 to 2.2-fold and only 1 upregulated (1.6-fold) (Table 2).

**Table 2:** Validated genes by qRTPCR

Parametric *P*-value	Fold-change	Gene symbol
2.90E-06	0.4457398	BCL11B
1.30E-06	0.4597414	DNMT1
2.50E-06	0.5009603	CDC25B
1.94E-05	0.5214546	CLSTN1
0.008539	0.5323253	VAMP5
1.81E-05	0.5435122	DOCK10
0.0005221	0.5693345	SPOCK2
0.0012085	0.5831974	ITGB7
0.001398	0.6124764	MCM3
0.0206755	0.6632639	CX3CR1
0.0026443	0.6749132	PTPN1
0.0034889	0.6847808	EP300
0.0125026	0.7141221	PPP2R1A
0.0184087	1.6090812	CLEC4D

Of the 47 considered genes, 14 were validated with significant *P*-values (*P *<0.05). q-RTPCR, quantitative reverse transcription polymerase chain reaction.

### Gene ontology analysis

Gene ontology (GO) analysis on the dataset showed two key immune-associated pathways to be altered, "negative regulation of adaptive immune response" and several ontologies affecting "thymic T cell selection" both with *P*-values <0.005 (Table 3).

**Table 3:** Gene ontology analysis

GO category	GO ontology	GO term	LS permutation *P*-value
GO:0002820	BP	Negative regulation of adaptive immune response	0.00001
GO:0033077	BP	T cell differentiation in the thymus	0.00001
GO:0043383	BP	Negative T cell selection	0.00001
GO:0045061	BP	Thymic T cell selection	0.00001

The dataset showed two key immune-associated pathways to be altered, "negative regulation of adaptive immune response" and several ontologies affecting "thymic T cell selection" both with *P*-values <0.005.

## Discussion

Gene expression profiling in disease reveals the underlying gene activity changes contributing to the disease process. This information provides insight into the tissue changes during the disease development and enables targets for therapeutic intervention to be identified. Secondly, strong, consistent gene expression changes can be utilized to generate diagnostic algorithms to identify early-stage disease before significant tissue damage has occurred.

However, in SpA, and in AS in particular, only a small number of case-control genomic profiling studies have been undertaken. The early studies involved low sample numbers and poor genome coverage and were very heterogeneous in terms of methodology, including the source of mRNA studied [16-19]. More recently four studies using genome-wide microarrays showed interesting results regarding SpA physiopathology and biomarker identification [20-23]. Of these, two were PBMC based [20,21] and in two the RNA was isolated from unfractionated peripheral blood [22,23]. However, all these studies did confirm that AS/SpA cases could be reliably distinguished from healthy controls using genomic profiling [20-23].

Although PBMCs have been widely used in autoimmune disease gene expression profiling studies, PBMCs do not represent an ideal tissue source for larger scale multicentre studies requiring extensive downstream processing soon after collection. PAXgene tubes enable whole blood to be collected directly into an RNA-preservative which stabilises the RNA from degradation for up to three days at room temperature, long enough for the samples to reach a safe frozen depository [9]. However, differences in the transcriptional profile between PBMC and PAXgene derived RNA have been demonstrated. These differences are due to the different cell populations targeted as well as the extra processing steps required for PAXgene samples to prevent globin mRNA overrepresentation affecting microarray sensitivity [9,24]. The study reported here has a number of strengths in that it represents a multicenter study involving ethnically homogeneous patients with AS (defined according to the modified New York criteria) and excluded patients on anti-TNF agents. Use of a primary dataset for candidate gene identification (by microarrays) followed by validation by qPCR in an independent dataset provides additional robustness.

Using the most variably expressed genes for unsupervised clustering gave us an estimate of the proportion of the gene expression variation driven by disease status. That only six controls and five AS samples were misclassified indicates the major driver of gene expression as disease status. However, the fact that not all samples classified correctly also indicates other factors driving gene expression variation. Such factors may be differences in blood collection, storage and transport protocols that can arise in multicentre studies. However, none of these effects were robust enough to cluster independently when tested (data not shown).

We validated 14 differentially expressed genes between AS patients and healthy controls. A number of these genes have well-documented inflammatory roles or an action on bone/cartilage metabolism. Moreover, GO analysis showed two key immune-associated pathways to be altered, "negative regulation of adaptive immune response" and several ontologies affecting "thymic T cell selection". Interestingly the GO analysis indicated a "negative" regulation of the immune system. This agrees with a previous expression profiling recently reported in PBMCs from AS patients [20] and is also consistent with the "reverse IFN gamma signature" reported by another group studying macrophages from AS patients [19].

A possible reduced immune response also correlates with the downregulation of *PTPN1 *and *DOCK10*, which are both involved in mediating IL4 actions [25]. Protein tyrosine phosphatase 1B (PTP1B) is a ubiquitously expressed enzyme shown to negatively regulate multiple tyrosine phosphorylation-dependent signalling pathways, including IL4 signalling [26]. Dock10 is also regulated by IL4 in B cells [27]. This is of particular interest as IL4 may play a role in AS pathogenesis. Interleukin 4 (IL4), a 20-kDa product from activated T lymphocytes, has a variety of stimulatory and inhibitory actions on B and T cells [25,28-30]. Recent studies have also indicated a potential role for IL4 producing CD8+ T cells in the pathogenesis of AS. Although CD8+ T cells are predominately associated with the production of 'Th1' cytokines, such as IFNgamma, there is now good evidence that some subsets of these cells can also produce 'Th2' cytokines such as IL4, IL5 and IL10 [31]. The potential functions associated with IL4-producing CD8+ T cells are as yet unclear but the subtype CD8+/TCR alpha beta + T cells, with a regulatory phenotype and function (expressing CD25+, CTLA4+, Foxp3+, but negative for IFNgamma and perforin), were previously described in peripheral blood of AS patients [32]. These results were confirmed in a recent study suggesting an altered pattern of CD8+ T cell differentiation in AS and in *HLA-B27*+ healthy individuals [33]. This predisposition to generate IL4+CD8+ T cells may play a role in pathogenesis of SpA [32,33].

In addition, the activation of the innate immune system has been proposed to play an important role in AS inflammation. Dysregulation of Toll-like receptor (TLR)-related pathways (an upregulation of TLR4 and TLR5), involved in innate immune response, have been described [23]. Interestingly we also identified increased expression of TLRs 4 and 5, together with TLR1 but the significance was marginal and, therefore, not followed up. However, an upregulation of C-type lectin domain family 4, member D (CLEC4D), another gene involved in the innate immune response, was seen. CLEC4D has been found to be expressed in a monocyte/macrophage restricted manner, and although no ligand or biological function has as yet been described, the receptor has been shown to be upregulated at the transcript level in a number of disease settings, similarly to two others members of the family, Mincle and Dectin-2. They are able to recognize and promote pathogen clearance and induce inflammatory signals [34]. This process seems to follow the Syk and caspase recruitment domain protein (CARD9) pathway which was recently implicated in a mouse model of SpA [35].

Changes in *SPOCK2 *(osteonectin) and *EP300 *provide interesting insights into AS progression. *SPOCK2*, also known as Sparc/osteonectin, was implicated, in a recent study, as a discriminator between SpA and healthy controls [22] and has been hypothesised to play roles in the regulation and production, assembly, or maintenance of matrix turnover in cartilage [36,37]. In this process TGFbeta and IFNgamma exert antagonistic effects, and play important roles in the physiologic regulation of extracellular matrix turnover. TGFbeta positively regulates *COL1A2 *through the cellular Smad signal transduction pathway, contrary to IFNgamma which downregulates *COL1A2 *through Stat1. Interestingly, the protein produced by EP300 belongs to the group of nuclear p300/CBP transcriptional coactivators for both Smad3 and Stat1a, and integrates signals that positively or negatively regulate *COL1A2 *transcription [38]. In addition, the downregulation of *EP300 *may promote a pro-inflammatory status [39] contributing to cartilage degradation. The protein phosphatase 2, regulatory subunit A (*PPP2R1A*) has also been shown to play a role in TGFβ-mediated regulation of Smad3-activated genes [40]. Finally, transactivated p300, controlled by phosphoinositide-3 kinase (PI3K)/AKT, is an important transcriptional co-activator of Sox9 [41], which modulates the expression of the major extracellular matrix component, aggrecan. Not surprisingly, altered *EP300 *expression has been associated with the Wnt pathway, a key mediator of bone formation as well as cartilage alterations in osteoarthritis [42]. Downregulation of these genes might lead to a loss of matrix integrity thereby accelerating tissue damage. PTP1B has also been shown to induce apoptosis in chondrocytes, thus downregulation might result in increased chondrocyte numbers contributing to joint damage as has been seen in osteoarthritis [43]. Both EP300 and DNA (cytosine-5-)-methyltransferase 1(*DNMT1*), mediate STAT3 functionality [44] which has also been associated in genetic studies with AS [45]. Decreases in STAT3 signalling might also contribute to the hypothesised reduction in immune response [46].

CX3CR1, which plays a pro-inflammatory role in RA, was also downregulated. In RA, CX3CR1, and its ligand CX3CL1, drives chemotaxis of pro-inflammatory monocytes to inflammatory sites [47]. Decreased expression of this gene in AS may reflect the fundamental differences in disease processes between AS and RA.

Beta 7 integrins have been shown to play a role in chronic ileitis [48,49] a common clinical feature of SPA. In our study, beta 7 integrin (*ITGB7*) is under-expressed again possibly reflecting a decreased immune response in AS.

The specific function of several other genes, as *BCL11B*, *CDC25B*, *VAMP5*, *MCM3*, *CLSTN1 *have not yet been determined and their potential involvement in disease processes needs additional research.

## Conclusions

We have validated a gene expression signature for AS from whole blood and identified strong candidate genes that may play roles in both the inflammatory environment and bone and cartilage effects. Future studies are needed to confirm some of the possible interactions suggested by this study.

## Abbreviations

AS: ankylosing spondylitis; BASDAI: Bath Ankylosing Spondylitis Disease Activity Index; BASFI: Bath Ankylosing Spondylitis Functional Index; BASMI: Bath Ankylosing Spondylitis Metrology Index; GO: gene ontology; mSASSS: modified Stoke Ankylosing Spondylitis Spinal Score; PBMCs: peripheral blood mononuclear cells; qPCR: quantitative polymerase chain reaction; q-RTPCR: quantitative reverse transcription polymerase chain reaction; SpA: spondyloarthritis; TLDA: TaqMan Low Density Array Cards.

## Competing interests

The authors declare that they have no competing interests.

## Authors' contributions

FMPS and GT participated in the conception and design of the study, carried out the lab work, performed the data analysis and drafted the manuscript. DL, HGP, JCB and MAB participated in the conception and design of the study and critically revised the manuscript. JEF critically revised the manuscript. FMPS, MM, AFM, JC, HS, AB, FG, PP and MC were involved in primary data collection. All authors read and approved the final manuscript.

## Supplementary Material

Additional file 1 **Supplementary Table S1: Characteristics of subjects involved in microarrays study**. BASDAI, Bath Ankylosing Spondylitis Disease Activity Index; BASFI, Bath Ankylosing Spondylitis Functional Index; BASMI, Bath Ankylosing Spondylitis Metrology Index; mSASSS, modified Stoke Ankylosing Spondylitis Spinal Score. Click here for file

Additional file 2 **Supplementary Table S2: Characteristics of subjects involved in TLDA study**. AS (*n *= 78) and healthy controls (*n *= 78). No significant differences for age and sex between groups. Click here for file

Additional file 3 **Supplementary Table S3: Genes differentially expressed between AS patients and controls by microarrays**. A total of 648 probes were considered significantly differentially expressed (80% confidence level of false discovery rate with 10% false positives). Click here for file
