# Supplementary material for: Whole blood transcriptional profiling in ankylosing spondylitis identifies novel candidate genes that might contribute to the inflammatory and tissue-destructive disease aspects
Source: Arthritis Res Ther. 2011 Apr 7;13(2):R57. doi: 10.1186/ar3309 (PMC3132052; doi:10.1186/ar3309)
Supplement: Additional file 1 — Supplementary Table S1: Characteristics of subjects involved in microarrays study. BASDAI, Bath Ankylosing Spondylitis Disease Activity Index; BASFI, Bath Ankylosing Spondylitis Functional Index; BASMI, Bath Ankylosing Spondylitis Metrology Index; mSASSS, modified Stoke Ankylosing Spondylitis Spinal Score. [file ar3309-S1.PDF]

Additional files

Supplementary Table 1

Title: Characteristics of subjects involved in microarrays study.

Legend: BASDAI - Bath Ankylosing Spondylitis Disease Activity Index; **BASFI** - Bath Ankylosing Spondylitis Functional Index; **BASMI** - Bath Ankylosing Spondylitis Metrology Index; **mSASSS -** modified Stoke Ankylosing Spondylitis Spinal Score.

Characteristics	AS Patients (n=18)
Male:Female (%)	10:8 (55.6:44.4)
Age (years; mean±SD) [range]	45.9±12.9 [21-64]
AS duration (years; mean±SD) [range]	16.0±12.9 [2-42]
Familiar history [n(%)]	5 (27.8)
BASDAI (mean±SD) [range]	5.9±1.3 [4.2-8.2]
BASFI (mean±SD) [range]	6.0±2.3 [4.1-8.2]
BASMI (mean±SD) [range]	4.6±2.9 [1-9]
mSASSS (mean±SD) [range]	17.3±21.5 [2-72]

BASDAI - Bath Ankylosing Spondylitis Disease Activity Index; **BASFI** - Bath Ankylosing Spondylitis Functional Index; **BASMI** - Bath Ankylosing Spondylitis Metrology Index; **mSASSS -** modified Stoke Ankylosing Spondylitis Spinal Score.
