# Supplementary material for: Whole blood transcriptional profiling in ankylosing spondylitis identifies novel candidate genes that might contribute to the inflammatory and tissue-destructive disease aspects
Source: Arthritis Res Ther. 2011 Apr 7;13(2):R57. doi: 10.1186/ar3309 (PMC3132052; doi:10.1186/ar3309)
Supplement: Additional file 2 — Supplementary Table S2: Characteristics of subjects involved in TLDA study. AS (n = 78) and healthy controls (n = 78). No significant differences for age and sex between groups. [file ar3309-S2.PDF]

Additional files

Supplementary Table 2

Title: Characteristics of subjects involved in TLDA study.

Legend: AS (n=78) and healthy controls (n=78). No significant differences for age and sex between groups.

Characteristics	Patients AS (n=78)
Male:Female (%)	45:32 (58.4:41.6)
Age (years; mean±SD) [range]	48.9±13.3 [21 - 73]
AS duration (years; mean±SD) [range]	21.9±14.8 [0-46]
Familiar history (n(%))	29 (37.7)
BASDAI (mean±SD) [range]	5.2±1.8 [1.7-9.6]
BASFI (mean±SD) [range]	5.0±2.5 [0.4-10]
BASMI (mean±SD) [range]	4.4±2.7 [0-9]
mSASSS (mean±SD) [range]	22.1±23.4 [1-72]
