# Supplementary material for: Whole blood transcriptional profiling in ankylosing spondylitis identifies novel candidate genes that might contribute to the inflammatory and tissue-destructive disease aspects
Source: Arthritis Res Ther. 2011 Apr 7;13(2):R57. doi: 10.1186/ar3309 (PMC3132052; doi:10.1186/ar3309)
Supplement: Additional file 3 — Supplementary Table S3: Genes differentially expressed between AS patients and controls by microarrays. A total of 648 probes were considered significantly differentially expressed (80% confidence level of false discovery rate with 10% false positives). [file ar3309-S3.PDF]

Additional files

Supplementary Table 3

Title: Genes differentially expressed between AS patients and controls by microarrays.

Legend: 648 probes were considered significantly differentially expressed (80% confidence level of false discovery rate with 10% false positives).

Gene symbol	Parametric p-value	Fold-change (AS/Cont)
CX3CR1	3.45E-04	0.58
LOC643031	8.47E-05	0.61
DGKQ	1.45E-05	0.62
HNRPR	6.78E-05	0.65
SPOCK2	3.07E-04	0.65
SEPT09	2.56E-04	0.66
SBK1	1.69E-04	0.66
CDC25B	2.00E-04	0.67
GZMM	3.39E-04	0.67
CLSTN1	4.53E-04	0.68
PTPN1	1.50E-06	0.69
DKFZp761P0423	4.71E-04	0.7
DOCK10	1.73E-04	0.7
EP300	1.26E-04	0.7
MAPK8IP3	3.37E-04	0.7
DKFZP434B0335	1.42E-04	0.71
DNMT1	6.46E-05	0.71
MGC3207	1.36E-05	0.71
PPP2R1A	1.73E-04	0.71
XPC	5.10E-06	0.71
CYFIP2	2.35E-04	0.72
IL27RA	4.40E-06	0.72

MYCBP2	3.63E-05	0.72
SFRS15	9.41E-05	0.72
AHNAK	4.47E-04	0.73
CTGLF3	2.87E-04	0.73
FAM62A	3.44E-04	0.73
MCM3	4.24E-04	0.73
MGC3207	5.25E-05	0.73
NUMA1	2.54E-04	0.73
RALGDS	4.39E-04	0.73
SEC31A	8.50E-05	0.73
ARHGEF18	4.95E-04	0.75
BRPF1	3.24E-04	0.75
SETD1A	9.84E-05	0.75
ZC3H4	4.39E-04	0.75
ADA	4.62E-04	0.76
BBX	9.55E-05	0.76
PTBP1	1.66E-04	0.76
PTBP1	3.85E-04	0.76
TMEM109	6.71E-05	0.76
CEP350	1.40E-04	0.77
LDOC1L	1.29E-04	0.77
MAPK7	1.54E-05	0.77
PAICS	9.59E-05	0.77
ALDH6A1	2.54E-04	0.78
BAGE5	3.14E-04	0.78
C17orf63	5.10E-06	0.78
GTF3C2	1.52E-05	0.78
LPXN	1.94E-04	0.78
LSS	9.80E-05	0.78
MMS19L	1.98E-04	0.78
TMEM80	2.05E-05	0.78
HELZ	1.08E-04	0.79
KCTD13	2.21E-04	0.79
NONO	2.78E-04	0.79
OSBP	7.05E-05	0.79

URG4	2.06E-04	0.79
IMAGE:4619448 5	4.28E-04	0.79
ACO1	4.85E-04	0.8
ALMS1	2.44E-04	0.8
CASP2	2.18E-04	0.8
ELMO2	1.16E-04	0.8
LOC647000	2.05E-04	0.8
NUP188	3.69E-04	0.8
RHOT2	1.87E-04	0.8
TH1L	3.56E-04	0.8
TH1L	3.81E-04	0.8
TNPO2	3.92E-04	0.8
UCKL1	4.69E-04	0.8
ATP2A2	4.73E-04	0.81
IL2RG	2.70E-04	0.81
METTL3	3.84E-04	0.81
PTPRC	2.55E-04	0.81
RNPS1	1.59E-04	0.81
ARHGEF7	1.82E-04	0.82
CDC2L5	2.32E-05	0.82
CLK2	5.78E-05	0.82
KLF2	3.43E-04	0.82
LY9	3.56E-04	0.82
SLC25A5	2.15E-04	0.82
TROVE2	6.10E-06	0.82
TSHZ1	4.47E-04	0.82
ANKRD17	2.06E-04	0.83
HOXC4	1.45E-04	0.83
IRF2BP2	3.95E-04	0.83
NSD1	1.06E-04	0.83
ODF2	4.55E-05	0.83
SFRS17A	4.62E-04	0.83
VPS52	3.14E-04	0.83
ALG8	1.19E-04	0.84
BBS1	2.13E-05	0.84

CDC2L5	7.68E-05	0.84
GOT1	2.67E-04	0.84
LOC650155	2.99E-04	0.84
MAP3K7IP1	4.56E-04	0.84
MED15	2.80E-04	0.84
XPNPEP1	3.62E-04	0.84
ZNF318	4.42E-04	0.84
DUSP14	1.18E-04	0.85
GTF2H4	4.26E-04	0.85
LOC401720	4.17E-05	0.85
ZNF609	4.19E-04	0.85
ADCY9	1.81E-04	0.86
BCOR	2.34E-04	0.86
FLJ41813	1.67E-04	0.86
IMAGE:5299888	1.69E-04	0.86
POLS	2.20E-04	0.87
TRIM32	7.15E-05	0.87
TAF1	2.56E-04	0.88
C5orf25	3.32E-04	0.89
KIAA0090	1.15E-04	0.89
SRCAP	3.62E-04	0.9
OCRL	2.61E-04	0.91
PARG	3.20E-04	0.91
IMAGE:2385542	8.69E-05	1.09
CETP	2.93E-04	1.1
POLR3F	4.51E-04	1.12
ASPH	8.96E-05	1.13
FAM103A1	2.95E-05	1.15
RNASE4	1.29E-04	1.16
CCNH	4.58E-05	1.17
CCDC89	4.02E-04	1.18
MYH4	2.89E-04	1.18
RPS7	4.06E-04	1.18
DCDC5	3.87E-04	1.19
ISCU	1.79E-04	1.19

HGD	7.50E-05	1.2
POLR2J3	4.60E-04	1.21
PRPF18	4.52E-04	1.21
C11orf67	3.90E-06	1.22
KIAA1160	2.34E-05	1.22
MYL6B	2.05E-04	1.22
BTBD7	5.00E-07	1.23
HSBP1	2.13E-04	1.23
COP1	1.04E-04	1.24
DPH3	1.03E-05	1.24
HOXB1	6.80E-06	1.24
RAG1AP1	2.78E-04	1.24
TM9SF3	3.69E-04	1.24
CYB5R1	3.29E-05	1.25
S100A4	1.19E-04	1.25
WBP5	4.50E-06	1.25
C12orf31	3.51E-04	1.26
LOC648638	5.02E-05	1.26
NUDT3	3.36E-04	1.26
S100A4	6.80E-06	1.26
COX5B	3.11E-04	1.27
HIST1H2BJ	7.56E-05	1.27
ARHGAP11A	5.84E-05	1.28
NSMCE2	2.86E-04	1.28
ARPC4	4.22E-04	1.29
NGFRAP1	8.43E-05	1.29
DDIT3	1.70E-04	1.3
TATDN1	3.61E-04	1.3
COX6A1	4.37E-04	1.31
HTATIP2	4.45E-04	1.31
LSM10	2.31E-05	1.31
TMEM97	1.47E-04	1.33
HNMT	2.92E-04	1.36
TNNC2	2.63E-05	1.36
MAPBPIP	3.50E-06	1.37

RPL34	8.63E-05	1.37
SCNM1	4.68E-04	1.38
ZMAT2	1.58E-04	1.38
MYL6	1.34E-04	1.39
PPP2R3C	2.62E-04	1.4
DBI	8.46E-05	1.41
ATP5EP2	1.85E-04	1.42
GMFG	3.91E-05	1.43
RPS24	2.88E-04	1.43
SLU7	3.56E-04	1.43
SYF2	4.80E-04	1.43
HNMT	1.63E-04	1.44
MYL6	2.50E-05	1.44
VAMP5	4.21E-04	1.44
NGFRAP1	2.90E-04	1.45
CKLF	2.75E-04	1.46
CKLF	4.30E-04	1.46
ATP5J	6.55E-05	1.48
SF3B14	2.66E-04	1.48
MRPL22	3.66E-04	1.51
CLEC4D	3.47E-04	1.52
UBL5	8.93E-05	1.52
LOC653147	3.63E-04	1.56
COP1	4.51E-04	1.57
HMGB2	5.77E-05	1.59
SF3B14	2.13E-04	1.61
UQCRB	2.33E-04	1.63
TXN	6.23E-05	1.67
TXN	2.54E-05	1.68
ATP5J	1.31E-04	1.71
ATP5J	8.43E-05	1.73
CMTM2	4.78E-04	1.74
CIP29	1.38E-04	1.75
PSMA4	8.45E-05	1.8
CHMP5	2.19E-04	1.83

NDUFB3	1.03E-04	1.85
PDCD10	4.58E-04	1.87
NDUFB3	1.48E-04	1.91
LOC651064	1.16E-04	1.96
LSM3	5.23E-05	2.14
GNG11	1.41E-04	2.15
CCDC72	9.64E-05	2.21
